# Perceptual and cognitive processes in augmented reality – comparison between binocular and monocular presentations

**DOI:** 10.3758/s13414-021-02346-6

**Published:** 2021-08-23

**Authors:** Akihiko Dempo, Tsukasa Kimura, Kazumitsu Shinohara

**Affiliations:** 1 Department of Systems Design Engineering, Faculty of Science and Technology, Seikei University, Tokyo, Japan; 2 The Institute of Scientific and Industrial Research (ISIR), Osaka University, Osaka, Japan; 3 Applied Cognitive Psychology Lab., Graduate School of Human Sciences, Osaka University, Osaka, Japan

**Keywords:** Binocular vision: Rivalry/ Bistable Perception, Attention: Selective, Physiological psychology

## Abstract

In the present study, we investigated the difference between monocular augmented reality (AR) and binocular AR in terms of perception and cognition by using a task that combines the flanker task with the oddball task. A right- or left-facing arrowhead was presented as a central stimulus at the central vision, and participants were instructed to press a key only when the direction in which the arrowhead faced was a target. In a small number of trials, arrowheads that were facing in the same or opposite direction (flanker stimuli) were presented beside the central stimulus binocularly or monocularly as an AR image. In the binocular condition, the flanker stimuli were presented to both eyes, and, in the monocular condition, only to the dominant eye. The results revealed that participants could respond faster in the binocular condition than in the monocular one; however, only when the flanker stimuli were in the opposite direction was the response faster in the monocular condition. Moreover, the results of event-related brain potentials (ERPs) showed that all stimuli were processed in both the monocular and the binocular conditions in the perceptual stage; however, the influence of the flanker stimuli was attenuated in the monocular condition in the cognitive stage. The influence of flanker stimuli might be more unstable in the monocular condition than in the binocular condition, but more precise examination should be conducted in a future study.

## Introduction

In the field of cognitive psychology, there are a great number of studies on the perceptual and cognitive differences between the binocular and monocular presentation of stimuli. For binocular presentation, better task performances are reported in some tasks, such as shorter reaction time, higher hit rate, and lower threshold, and this phenomenon is known as “binocular summation” (Baker et al., 2018; Blake & Fox, 1973; Eriksen et al., 1966). Another example of the difference between binocular and monocular presentation is “binocular rivalry.” This is a phenomenon in which perception in the observer’s mind changes mutually and ongoingly when completely different stimuli are presented to each eye (Chong et al., 2005; Chong & Blake, 2006; Hancock & Andrews, 2007; Kovács et al., 1996; Levelt, 1966; Song & Yao, 2009; Zhang et al., 2011). These phenomena reveal that perception and cognition differ between binocular and monocular presentation.

This difference might be influential in actual use cases of augmented reality (AR). AR is an emerging technology in which information is superimposed onto the real world directly (Azuma, 1997; Azuma et al., 2001; Chatzopoulos et al., 2017). AR is expected to improve the usability and visibility of information that is typically presented on a display in conventional methods (Castillo & Olga, 2016; Dixon et al., 2013; Rusch et al., 2013; Schömig et al., 2018; Schwarz & Fastenmeier, 2017).

Two types of methods are used in AR systems: binocular presentation and monocular presentation (Bayle et al., 2019; Kitamura et al., 2014, 2015; Kitamura et al., 2019; Sasaki et al., 2010). With binocular AR, information is presented to both eyes of the user, and with monocular presentation, it is presented to only one of their eyes. Therefore, perceptual and cognitive differences might influence the actual usability.

Some previous studies have reported the differences between binocular and monocular presentation in AR use. Kitamura et al. (2015) reported that monocular AR leads to higher accuracy in a tracing task compared with binocular AR when an AR image covered the real world. Furthermore, Kitamura et al. (2019) investigated “change blindness” in AR to compare binocular presentation and monocular presentation. Change blindness is a phenomenon that occurs when two slightly different images are presented sequentially and a distractor stimulus is presented between them, and the observer often overlooks the change between the two images (Jensen et al., 2011; Rensink et al., 1997). In Kitamura et al. (2019), the distractor was an AR image that was presented binocularly or monocularly. The results revealed that participants could detect the change in the monocular condition as fast as when the distractor was not presented, revealing that change blindness had not occurred; however, a much longer duration was needed to detect the change in the binocular condition.

Thus, monocular AR has advantages for tasks conducted in the real world. The reason for the advantages reported in the previous studies might be explained as follows. With monocular AR, users might be able to choose the information required for a task in the stage before the information from each eye is integrated because AR information is presented to only one of their eyes. As a result, they might be able to observe the real world by using the eye to which AR information is not presented. However, with binocular AR, users might not be able to avoid the AR information because the information is presented to both eyes. This explanation may be consistent with the results of the previous studies mentioned above; however, this is only conceptual, not directly investigated. Therefore, there are insufficient data to support this idea, and more precise investigation is required.

Hence, in the present study, the difference between binocular and monocular AR was investigated by measuring physiological, behavioral, and subjective data to clarify the stage at which differences in perception or cognition between binocular and monocular presentation appear. For this purpose, we used a task that combines the flanker task with the oddball task.

In the typical flanker task, target and non-target stimuli (e.g., < and > , S and H) are presented in the central vision as central stimuli, and participants are asked to respond depending on the stimulus (Eriksen & Eriksen, 1974; Eriksen & Eriksen, 1979; Noyce & Sekuler, 2014; Sidarus & Haggard, 2016). At the same time, flanker stimuli are arranged at the sides of the central stimulus. In the congruent condition, the flanker stimuli’s direction and letter are the same as the central target (<<<<<, SSSSS), and in the incongruent condition, they are the opposite (>><>>, HHSHH). Participants must allocate their attention to the central stimulus; however, their attention includes some spatial width. If the flanker stimuli are inside the area to which attention is allocated, they are inevitably processed, resulting in a faster and more accurate response in the congruent condition and a slower and less accurate response in the incongruent condition. If the flanker stimuli are outside of the area, this effect is not observed.

In the flanker task in the present study, the participants respond only to the target, so they have to stop themselves from responding to the non-targets. To investigate cognitive processing in responding to targets and suppressing the response to non-targets, previous studies measured electroencephalograms (EEG) and analyzed event-related brain potentials (ERPs) in a Go/No-go task. In this case, as a physiological indicator, Go P3 at the central to parietal regions (i.e., Cz to Pz electrodes) is evoked for targets, and No-go P3 at the frontal to central regions (i.e., Fz to Cz electrodes) is evoked for non-targets (Pfefferbaum et al., 1985; Pfefferbaum & Ford, 1988). Furthermore, as a competitive reaction occurs between a central stimulus and flanker stimuli in the incongruent condition, N2 at Fz to Cz electrodes is evoked (Kopp, Mattler, et al., 1996a; Kopp, Rist, & Mattler,1996b).

In the typical oddball task, the same stimulus is repeatedly presented, and sometimes target and deviant stimuli are presented at a low probability. These rare target and deviant stimuli attract attention, resulting in P3s being evoked (Katayama & Polich, 1996, 1998). In summary, these ERPs reflect cognitive processing such as for stimulus conflict and attention.

In addition, manipulating the frequency of flanker stimuli by combining the flanker and oddball tasks makes it possible that a flanker stimulus will be treated as a deviation stimulus from the stimulus sequence. One previous study reported that low-probability flanker stimuli (i.e., deviation stimuli) elicit visual mismatch negativity (vMMN; Noyce & Sekuler, 2014). vMMN is caused by deviation from the regularity of sequential visual stimuli (e.g., Czigler, 2007; Pazo-Alvarez et al., 2003). Moreover, vMMN reflects the pre-attentional stage and occurs independently of attention (e.g., Stefanics et al., 2011). Therefore, this task is useful for revealing the difference between binocular/monocular visual information processing because the pre-attentional stage (vMMN) and cognitive stage (P3s) can be investigated by analyzing these ERPs in combination with flanker and oddball tasks. In the present study, arrowhead stimuli (< or >) were presented in the central vision repeatedly as a central stimulus, and one of them was treated as a target. Participants responded to the target by pressing an assigned key. In a small number of trials, flanker arrowheads were presented aside a central stimulus (e.g., <<<<<). These flanker stimuli were presented as AR images monocularly or binocularly, and participants were instructed to ignore the stimuli and focus only on the central stimulus to decide whether to press the key.

Using this task, we investigated whether, in the monocular condition, participants could respond to the required information and ignore the interfering stimuli at an early stage (i.e., perception). We hypothesized that participants could ignore information from the eye to which AR flanker stimuli were presented in the perceptual stage. If they could focus on only the required information in the monocular condition, it would mean that the flanker stimuli might not prevent the central stimulus from being seen; therefore, there might be no difference in reaction time between the flanker condition and the void condition, in which no flanker is presented.

In addition, if the hypothesis is true, the flanker stimuli would not be processed in the monocular condition; therefore, vMMN would not be evoked in the flanker as well as the void condition. vMMN would be evoked in the flanker condition only in the binocular condition, in which the flanker stimuli would inevitably be processed. Furthermore, in the monocular condition, N2 would not be evoked in the incongruent flanker condition as well as the void condition because the flanker itself would not be processed, resulting in reaction competition not occurring. In comparison, in the binocular condition, N2 would be evoked in the incongruent condition. Last, in the monocular condition, No-go P3 and Go P3 would be similar in the flanker condition to the void condition, and, in the binocular condition, the flanker stimuli would influence P3.

However, contrary to the assumption that the flanker stimuli would not be processed in the monocular condition, there is enough room to assume that the flanker stimuli would be processed not only in the binocular condition but also in the monocular condition because once the light of stimuli hits the retina, the stimuli are processed to some degree, though a specific percept might not be produced. In this case, it might be possible for ERPs to also be evoked in the monocular condition and that each ERP could be comprehended as below. If vMMN were evoked in the monocular condition as well as in the binocular condition, it would reveal that at least the flanker stimuli would be processed at the perceptual stage in both conditions. Moreover, if there were a difference between the monocular and binocular conditions for N2, it would reveal that the cognitive process is different between the two conditions at the stimuli evaluation stage. Last, if there were a difference between the monocular and binocular conditions for P3, it would reveal that the attentional cognitive process for selecting a reaction would be different between the two conditions.

## Method

This experiment was approved by the Behavioral Research Ethics Committee of the Osaka University School of Human Sciences. We acquired written informed consent from all of participants before the experiment started.

### Participants

Six students at Osaka University and 16 people arranged by a worker dispatch company participated in the experiment. Four of the participants were excluded from the analysis because of mechanical malfunction, misunderstanding the instructions, and low quality of data for ERPs, that is, the number of trials available for analyzing of P300 amplitude was less than 20 (Cohen & Polich, 1997). Data acquired from the 18 participants (male = nine, female = nine) were used in the analysis, and their mean age was 21.89 years (*SD* = 1.79). All participants had normal or corrected-to-normal vision (at least 0.7 in binocular decimal visual acuity). Nine participants had a right dominant eye, and nine had a left dominant eye. By using Ishihara color test II (24 plates), all participants were confirmed to have normal color vision.

### Apparatus and recording

**Fig. 1:**
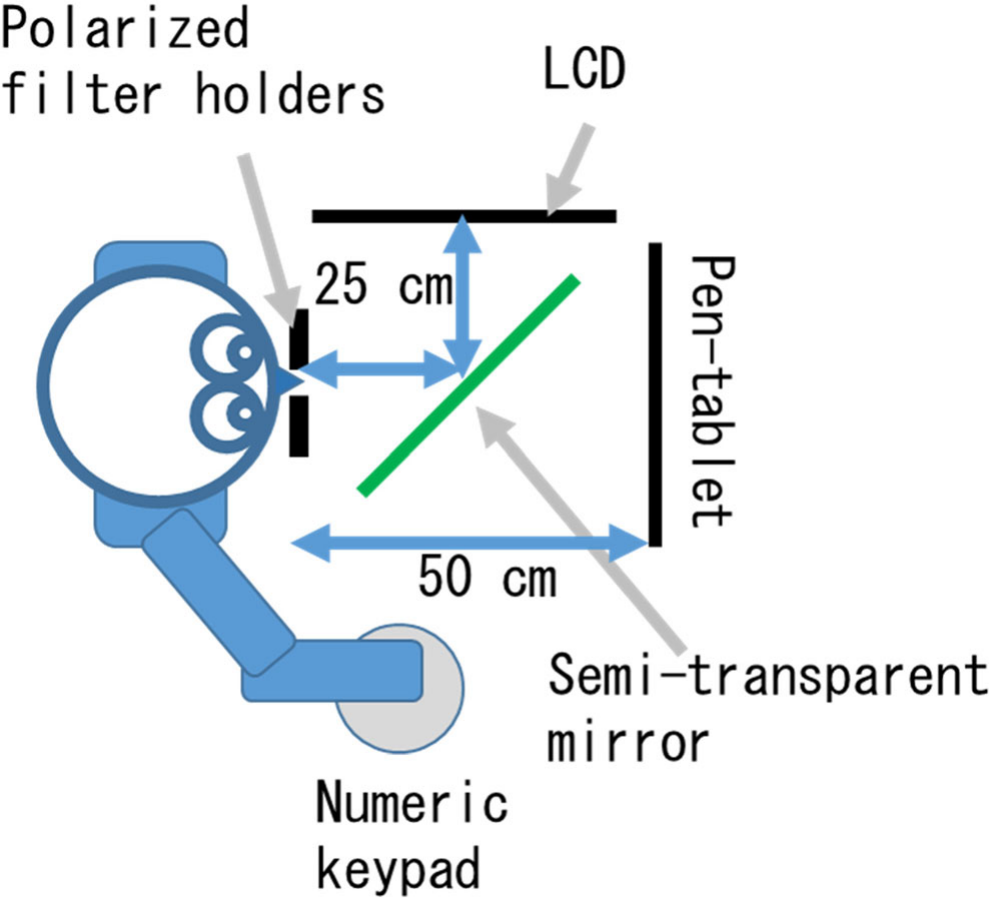
Arrangement of the apparatus. The image on the LCD monitor was reflected by a semi-transparent mirror so that it was presented as an augmented reality (AR) image in front of participants at the same distance as the pen-tablet monitor. By rotating polarized filters, the image could be presented to one or both eyes, i.e., binocularly or monocularly. Under the monocular condition, the AR image was presented to participant’s dominant eye

The experimental apparatus comprised polarized filter holders (Sigma Koki, PH-50), a semi-transparent mirror, a pen-tablet monitor (Wacom, Cintiq 22HD, resolution:1,680 × 1,050), an LCD (Mitsubishi, RDT235WX(BK), AX220 model, resolution: 1,920 × 1,080), a computer (Mouse computer, m-Book P500X1-M2SH2, OS: Windows 10), and a numeric keypad (Archisite, ASTKP1601). Figure 1 shows the arrangement. Programs for displaying stimuli and measuring responses were created by using Microsoft Visual Studio 2017.

EEG data were recorded by using Polymate AP1132 (Miyuki Giken, Japan) and an electrode cap (Easycap GmbH, Germany) using Ag/AgCl electrodes at 26 sites (Fp1, Fp2, F7, F3, Fz, F4, F8, FC3, FCz, FC4, T7, C3, Cz, C4, T8, CP3, CPz, CP4, P7, P3, Pz, P4, P8, O1, Oz, O2) according to the modified 10–20 System. In addition, electrodes were also placed on both earlobes (A1 and A2). The reference electrode was on the tip of the nose, and the ground electrode site was AFz. The data from all channels were recorded using the Mobile Acquisition Monitor Program (Miyuki Giken, Japan). The electrode impedances were kept below 10 kΩ. A DC filter was used during recording. The sampling rate was 1,000 Hz.

### Stimulus

**Fig. 2:**
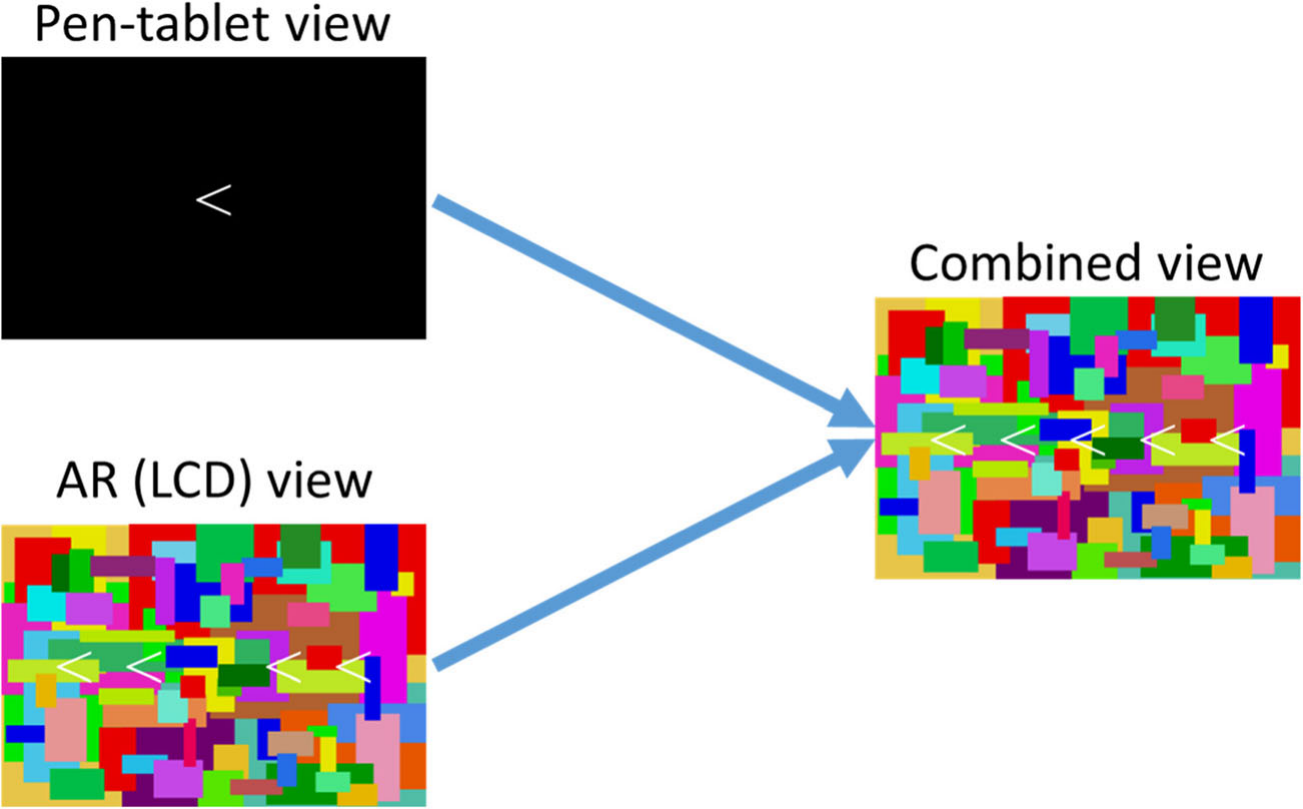
Stimuli and their arrangement. This figure shows the congruent condition, which included presentation of four arrowheads in center of an augmented reality (AR) image. In the void condition, there were no arrowheads in the AR image. In the incongruent condition, arrowheads in the AR image were in the opposite direction. For better visibility, arrowheads are emphasized in terms of size, thickness, and contrast

Right- and left-facing arrowheads (< >) were used as target, non-target, and flanker stimuli (see Fig. 2). The size of each stimulus was 0.5° of visual angle squared. In most of the trials, only one arrowhead was presented at the center of the pen-tablet monitor. In some trials, five were displayed side by side, only the central stimulus was displayed on the pen-tablet monitor, and the others were presented as an AR image. The distance between the center of each stimulus was 0.5° in visual angle. Before each block started, an experimenter presented cross marks (+) on both the LCD and the pen-tablet monitor. Then, participants adjusted the location of the cross mark on the pen-tablet monitor so that the cross marks could be seen overlapping at the same location. Hence, as seen from the participants, the central coordinates of the LCD and pen-tablet monitor were at the same location, so the arrowheads on the LCD and pen-tablet monitor did not overlap with each other.

To emphasize the difference between the binocular and monocular presentation of AR, an AR Mondrian stimulus (an image that has various colors and numerous edges) covered all stimuli. The size of the AR Mondrian stimulus was 8.8° high × 13.2° wide.

### Procedure

**Fig. 3:**
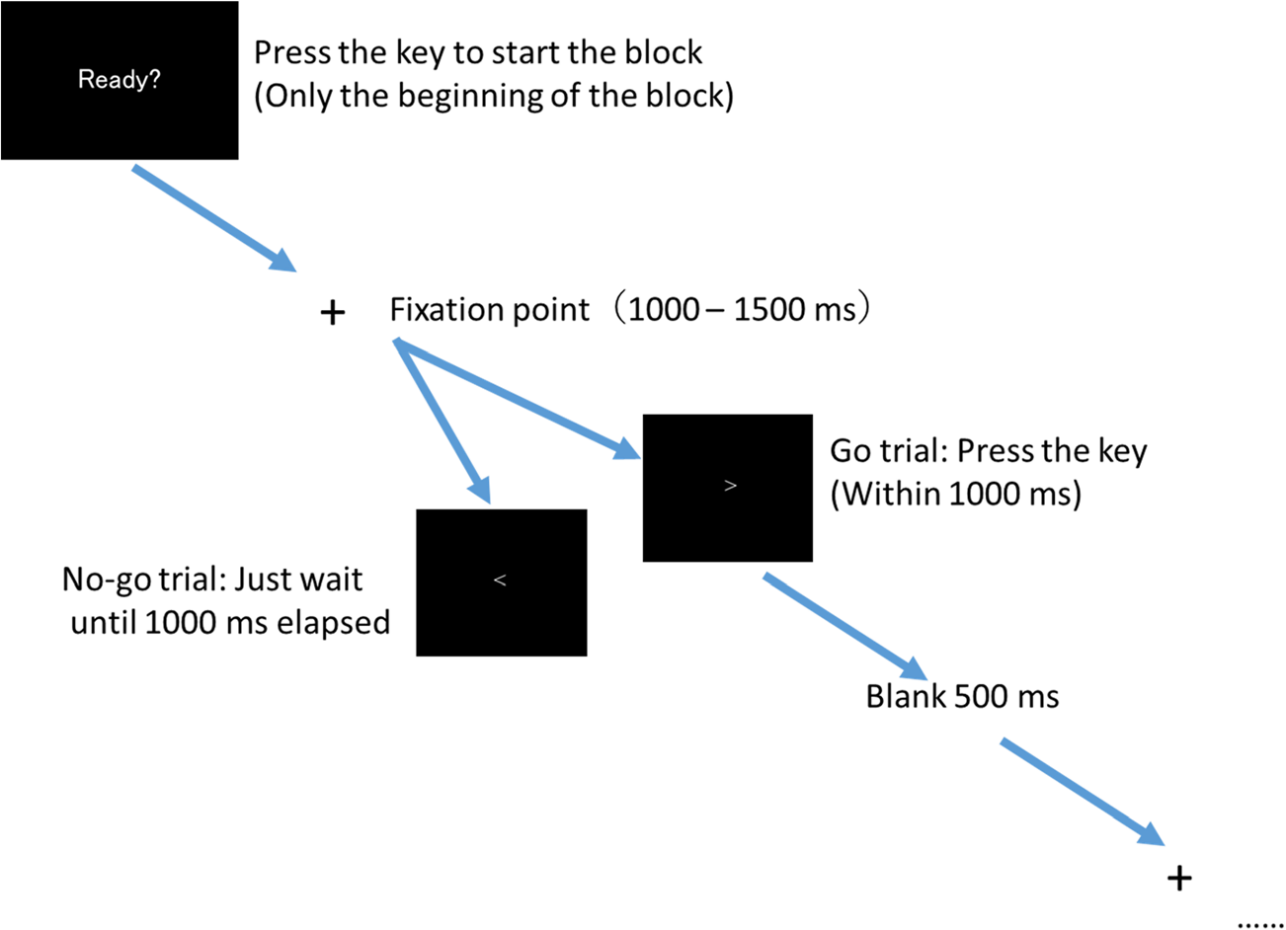
Experimental procedure. An augmented reality (AR) Mondrian stimulus always covered the display. Flanker stimuli were presented as an AR image in 20% of trials

Figure 3 shows the experimental procedure. First, the word “Ready?” appeared at the center of the pen-tablet monitor. Then, a participant pressed the 5 key on the numeric keypad, a cross was presented as a fixation point (duration: 1,000–1,500 ms at random), and an arrowhead followed (duration: 1,000 ms fixed). A blank screen was then presented for 500 ms; after that, the fixation point and the arrowhead were presented in the same way repeatedly. The direction of the arrowhead was right or left, one of which was assigned as a Go-target. Which direction became the Go-target was randomized between the participants. If the arrowhead was the Go-target, participants pressed the 5 key, and if not, they did not press anything and instead waited until the arrowhead disappeared. In 80% of trials, only one arrowhead was presented at the center of the display (void condition), and in the remaining 20%, the four flanker stimuli were presented next to the target (two of them on the left side, the other two on the right side of the central arrowhead; see Fig. 2). There were two directions in which the flanker stimuli faced: one was in the same direction as the central arrowhead (congruent condition), and the other was the opposite direction (incongruent condition). The flanker stimuli were presented as an AR image monocularly or binocularly, depending on the observation condition. Therefore, in the binocular condition, participants could see the flanker stimuli with both eyes; however, in the monocular condition, the flanker stimuli were presented to participants’ dominant eye. The participants were instructed that they had to decide whether to press the 5 key for the central arrowhead and ignore the flanker stimuli. In addition, the AR Mondrian stimulus covered the background throughout the trials except when “Ready?” was presented.

After 120 trials were finished, “Ready?” was presented again. During this section, participants were free to take a break and press the 5 key to start the next trial. These 120 trials were treated as a block, and five blocks were conducted sequentially for one observation condition. After five blocks were finished, an experimenter changed the observation condition, and the next five blocks then started. The order of observation conditions was randomized between participants. Before the monocular condition started, participants were instructed to direct their attention to the eye to which the AR image was not presented.

Between the fifth and sixth blocks (i.e., between the observation conditions), the participants answered a questionnaire orally (see Table 1). After the monocular condition, they answered Q1 to Q3, and after the binocular condition, they answered Q4 and Q5. The answers ranged from 1 (not at all) to 10 (very).

**Table. 1:** Contents of the questionnaire

No.	Question
Q1	You were instructed to direct your attention to the eye to which the AR image was not presented in the monocular condition. Subjectively, how well did you direct your attention as instructed?
Q2	How much was the flanker stimuli perceived in the monocular condition?
Q3	How much did the flanker stimuli annoy you in the monocular condition?
Q4	How much was the flanker stimuli perceived in the binocular condition?
Q5	How much did the flanker stimuli annoy you in the binocular condition?

### Flow of experiment

First, written informed consent was acquired from the participants. Next, the experimenter acquired demographic data from the participants and confirmed their dominant eye, decimal visual acuity, binocular vision, and color vision. After that, the experimenter explained the experimental procedure to the participants. Participants were told not to blink when pressing the key and to ignore the flanker stimuli. After instructions were given, the physiological measuring devices for EEGs were fitted onto the participants, the participants reconfirmed the experimental procedure, and the task was started.

### Experimental design

The number of trials was determined in accordance with the following formula: observation condition (2: monocular, binocular) × flanker condition (3: 48 void trials, 6 congruent trials, 6 incongruent trials) × target condition (2: Go, No-go) × repeat 5 blocks, equaling 1,200 trials. All factors were within-participant.

### Objective variables

As a subjective measure, the answers to the questionnaire were recorded (Table 1).

As a behavioral measure, the reaction time to the Go-target was recorded. In addition, the response to each target (whether the 5 key was pressed or not) was recorded. In a Go trial, pressing the button was the correct response, and in the No-go trial, the opposite was correct.

As a physiological measure, the mean amplitudes of ERPs were analyzed from EEGs. To analyze the data, the EEGLAB toolbox (Delorme & Makeig, 2004) and ERPLAB toolbox (Lopez-Calderon & Luck, 2014) for MATLAB (MathWorks Inc, Natick, MA, USA) were used. The data were digitally band-pass filtered at 0.1–30 Hz (6 dB/octave) using an IIR Butterworth analog simulation filter. Artifacts derived from eye movements and eye blinks were rejected using an automatic EEG artifact detector based on the joint use of spatial and temporal features (ADJUST) of the EEGLAB toolbox (Mognon et al., 2011). To extract vMMN, N2, and P3s, the EEG epoch was set at 1,000 ms (including a 200-ms pre-stimulus baseline). Epochs in which the EEG signal variation exceeded ± 100 μV were excluded from averaging as electrical noise caused by the activity of electromyograph. After artifact rejection, the numbers of remaining trials ranged as follows in the binocular condition, that is, Go void: 219–240 (0–8.7% of trials were rejected, Go congruent: 25–30 (0–16.6% of trials were rejected), Go incongruent: 27–30 (0–10.0% of trials were rejected), No-go void: 218–238 (0.8–9.2% of trials were rejected), No-go congruent: 27–30 (0–10.0% of trials were rejected), and No-go incongruent: 26–30 (0–13.3% of trials were rejected). For the monocular condition, the numbers were Go void: 224–240 (0–6.6% of trials were rejected), Go congruent: 28–30 (0–6.6% of trials were rejected), Go incongruent: 25–30 (0–16.6% of trials were rejected), No-go void: 217–238 (0.8–9.6% of trials were rejected), No-go congruent: 24–30 (0–20.0% of trials were rejected), and No-go incongruent: 25–30 (0–16.6% of trials were rejected). The time range for vMMN was defined as 180–320 ms, and N2 was defined as 400–500 ms. For the Go P3 and No-go P3, the time ranges were defined as 360–600 ms except for the incongruent condition in the binocular condition (incongruent condition in the binocular condition: 460–660 ms). These time ranges were decided on the basis of the peak latencies of the grand averaged waves set to contain the maximum ERP amplitude for each condition. The mean ERP amplitudes were calculated by averaging the data points (amplitudes) within each time range (Luck, 2014).

## Results

### Behavioral data analysis: Reaction times and hit rates

Participants were instructed to press the key only in the Go trial; hence, the reaction times in Go trials were used for analysis. The upper limit of reaction time was 1,000 ms because the arrowheads were presented for 1,000 ms. If participants could not respond to a Go-trial target within 1,000 ms, this was treated as a miss trial, and the data of the trial were not included in the analysis of reaction time. If the reaction time was less than 100 ms, it was treated as a premature reaction and also excluded.

The reaction time data were analyzed by using a linear mixed model. The objective variable was the reaction time, and the explanatory variables were the observation condition, flanker condition, and their interaction. Participants were treated as a random effect. A normal distribution for the error structure and the identity link function were used. The binocular condition and the void condition were treated as a reference category.

**Fig. 4:**
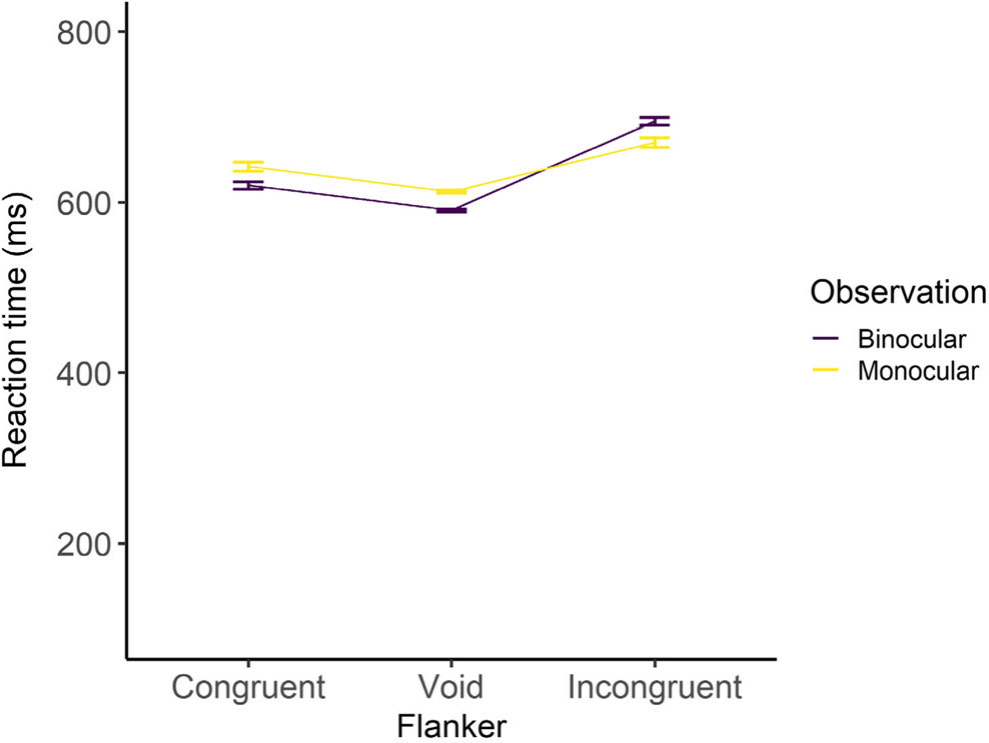
Reaction times in Go trials. Error bars indicate standard error

**Fig. 5:**
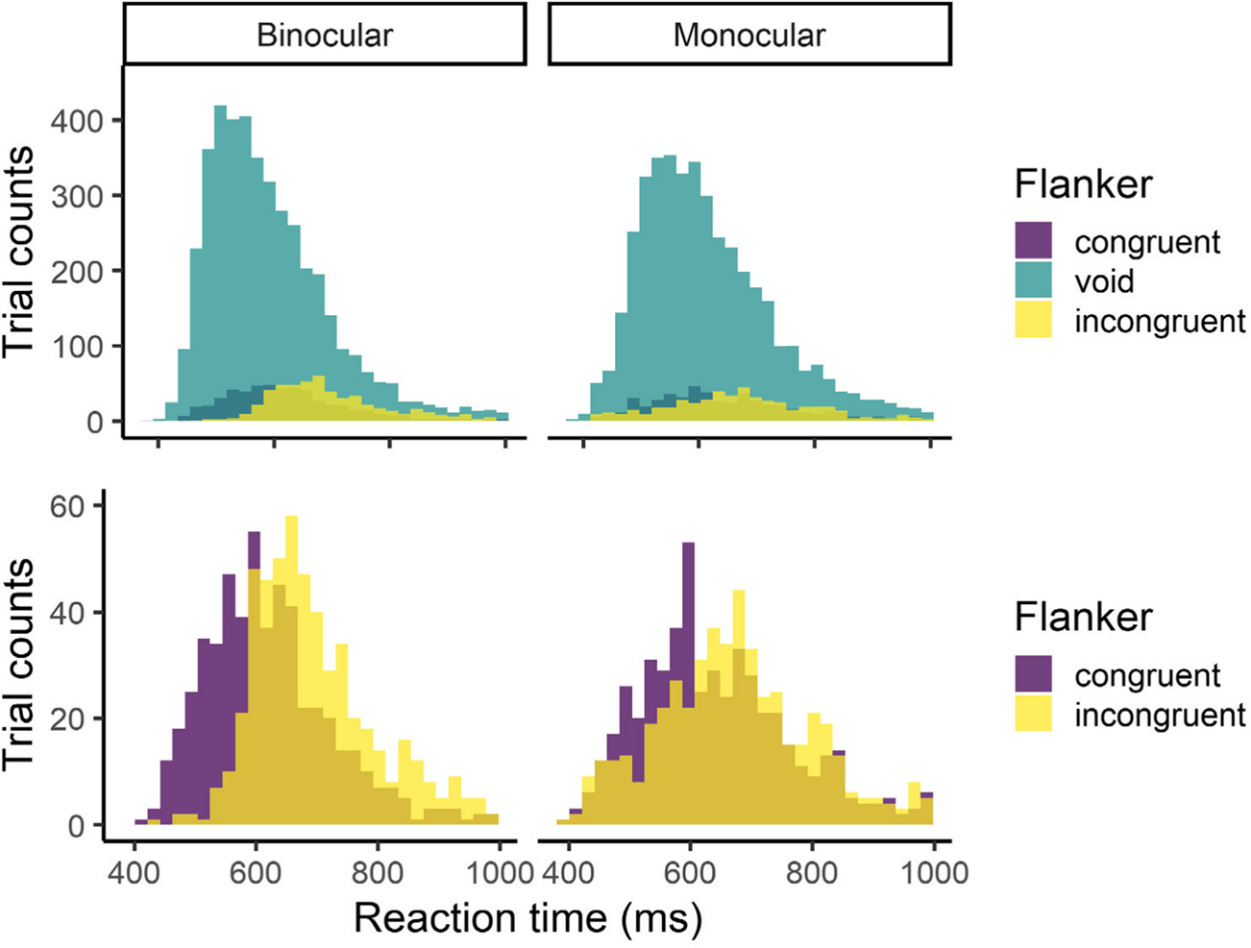
Histograms of reaction times. The upper figure includes all flanker conditions. For better visibility, the void condition was excluded in the lower figure. Note that scales of y axes are different between the upper and lower figures

The results are shown in Figs. 4 and 5 and Table 2. The effect of the observation condition was significant, and it was revealed that the reaction time was longer in the monocular condition than in the binocular condition. The effect of the flanker condition was also significant, and it was revealed that the reaction times were longer in both the congruent and the incongruent conditions than in the void condition. In addition, the interaction was significant, revealing that, in only the incongruent condition, the reaction time was shorter in the monocular condition than in the binocular condition. In summary, in both the congruent and incongruent conditions, the flanker stimuli prolonged the reaction time, and, basically, the reaction time became longer in the monocular condition; however, in only the incongruent condition, it was shorter compared with the binocular condition.

**Table. 2:** Analysis results for reaction time

	Estimate	*SE*	*t* value	*p* value
Intercept	592.423	15.351	38.592	< .001 ***
Bino vs. Mono	22.719	2.031	11.188	< .001 ***
Void vs. Con	28.228	4.284	6.589	< .001 ***
Void vs. Inc	105.058	4.321	24.316	< .001 ***
Mono/Con	2.941	6.116	0.481	.631
Mono/Inc	-46.912	6.176	-7.596	< .001 ***

AIC = 123813, BIC = 123871

Bino and Mono represent observation conditions (binocular and monocular, respectively). Void, Con (congruent), and Inc (incongruent) represent flanker conditions. Mono/Con and Mono/Inc represent interactions

We analyzed the hit rate data in each condition by using a generalized linear mixed model (GLMM). In general, d’ and the criterion based on the signal detection theory (Gescheider, 1997) are often used for analyzing Go/No-go tasks (Redick et al., 2011; Schulz et al., 2007); however, in the present study, the number of trials was different between the void condition and the congruent/incongruent conditions. When the hit rate or miss rate are 0% or 100%, these figures must be converted to calculate d’ and criterion. One common method is to convert the proportion of 0 to 1/(2N) and 1 to 1–1/(2N). N means the number of trials. Using this conversion, the higher the number of trials becomes, the higher the figure becomes after transformation of 100% (Huang & Ferreira, 2020). Therefore, in the present study, if we had used d’ inappropriately, the void condition, which had a higher number of trials, would have had an advantage compared with the other flanker conditions. Hence, we chose to use the hit rate as the objective variable.

Basically, the method used for the analysis was same as that for the reaction time, but a binomial distribution for the error structure and logit link function were used. A hit was coded as 1, and a miss was coded as 0.

**Fig. 6:**
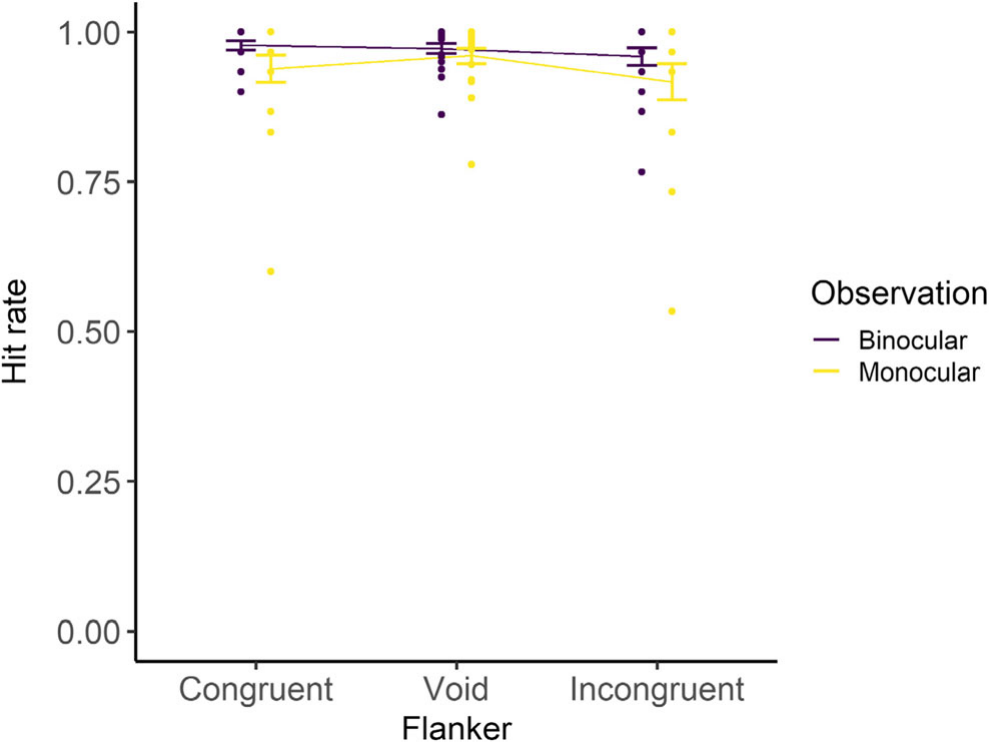
Hit rates for Go trials. Error bars indicate standard error. Dots indicate hit rates of each participant

The results are shown in Fig. 6 and Table 3. The effect of the observation condition was significant, revealing that the hit rate was higher in the binocular condition than in the monocular condition. The effect of the flanker condition was marginally significant in the incongruent condition. In addition, the interaction between the monocular and congruent conditions was marginally significant.

In summary, the binocular condition had a higher hit rate compared with the monocular condition, and an incongruent flanker might cause the hit rate to deteriorate. Furthermore, in the monocular condition, the hit rate might deteriorate not only in the incongruent condition but also in the congruent condition.

**Table. 3:** Analysis results for hit rates

	Estimate	*SE*	*z* value	*p* value
Intercept	4.205	0.299	14.04	< .001 ***
Bino vs. Mono	-0.405	0.124	-3.27	.001 **
Void vs. Con	0.221	0.310	0.71	.476
Void vs. Inc	-0.437	0.242	-1.81	.071 ^+^
Mono/Con	-0.715	0.350	-1.93	.054 ^+^
Mono/Inc	-0.428	0.303	-1.41	.158

AIC = 2976, BIC = 3027

Bino and Mono represent observation conditions (binocular and monocular, respectively). Void, Con (congruent), and Inc (incongruent) represent flanker conditions. Mono/Con and Mono/Inc represent interactions

### Physiological data analysis: vMMN, N2, No-go P3, and Go P3

The mean amplitude of vMMN at O1, where the vMMN was elicited at maximum amplitude, was assessed with a three-way repeated-measures analysis of variance (ANOVA; two observation conditions × three flanker conditions × two target conditions). The ANOVAs for the analysis of all ERPs were conducted by applying Greenhouse-Geisser corrections to the degrees of freedom (Greenhouse & Geisser, 1959) when Mauchly’s sphericity test was significant. The effect sizes are indicated in terms of partial eta squared (η_p_^2^). Post hoc comparisons were made using Shaffer’s modified sequentially rejective multiple test procedure, which extends Bonferroni t tests in a stepwise fashion (Shaffer, 1986). The significance level was set at *p* < .05 for all statistical analyses.

**Fig. 7:**
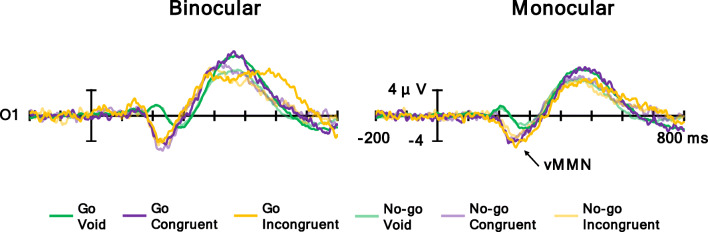
Grand averaged waves at O1

**Fig. 8:**
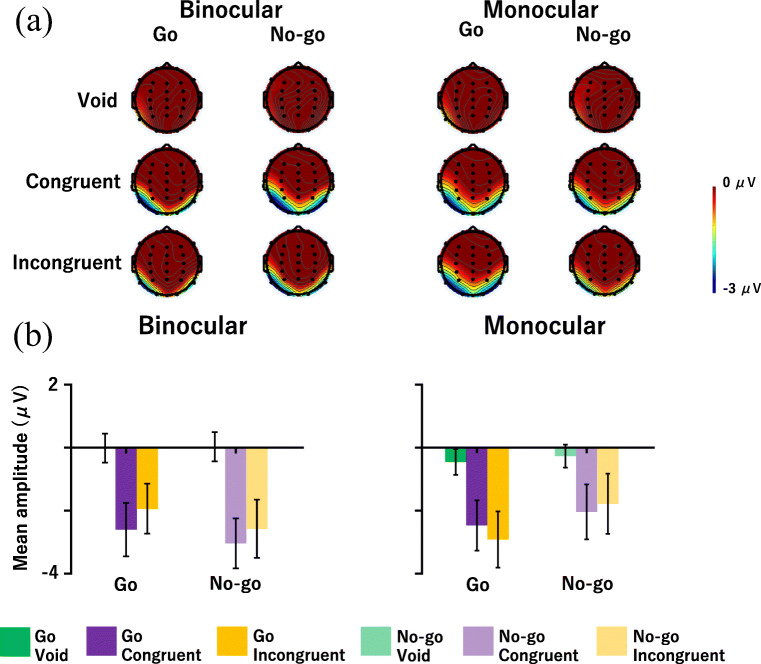
**a** Topographic maps for the time range of vMMN (180–320 ms) and **b** vMMN mean amplitude at O1. Error bars indicate standard error

Figure 7 shows the grand averaged waves at O1, and Fig. 8 shows (a) a topographic map for the time range of vMMN (180–320 ms) and (b) the vMMN mean amplitude at O1. vMMN is a negative potential; thus, the more negative the potential is, the larger the vMMN response is. The ANOVA results are summarized in Table 4. It was revealed that the main effect of the flanker condition was significant. Post hoc comparisons showed that the mean amplitudes of the congruent and incongruent conditions were larger in the negative direction than in the void condition (*p*s < .05). Moreover, the interaction of the observation and target conditions was significant. Post hoc comparisons indicated that the mean amplitude of the Go condition was larger in the negative direction than the No-go condition in the monocular condition (*p* < .05). The other main effects and interactions were not significant. In summary, the flanker stimuli elicited vMMN in both conditions, and the amplitude of vMMN was different between the Go and No-go conditions in the monocular condition.

**Table. 4:** Analysis results for vMMN

Interaction and main effect	*F*	*df*	*p*	η_p_^2^
observation × flanker × target	1.46	2, 34	.25	.08
observation × flanker	1.67	2, 34	.20	.09
observation × target	10.07	1, 17	.006^**^	.37
flanker × target	.18	2, 34	.81	.01
Observation	.01	1, 17	.91	.01
Flanker	16.76	2, 34	< .001^***^	.50
Target	.41	1, 17	.53	.02

The mean amplitudes of N2 for the Go condition at Fz, Cz, and Pz were assessed with a three-way repeated-measures ANOVA (two observation conditions × three flanker conditions × three electrodes (Fz, Cz, and Pz)) in each target condition to eliminate the influence of Go P3 and No-go P3.

**Fig. 9:**
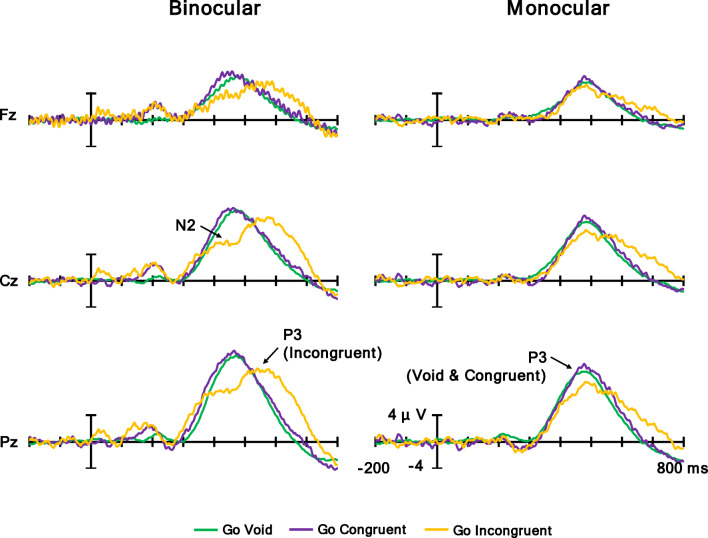
Grand averaged waves of Go condition at Fz, Cz, and Pz

**Fig. 10:**
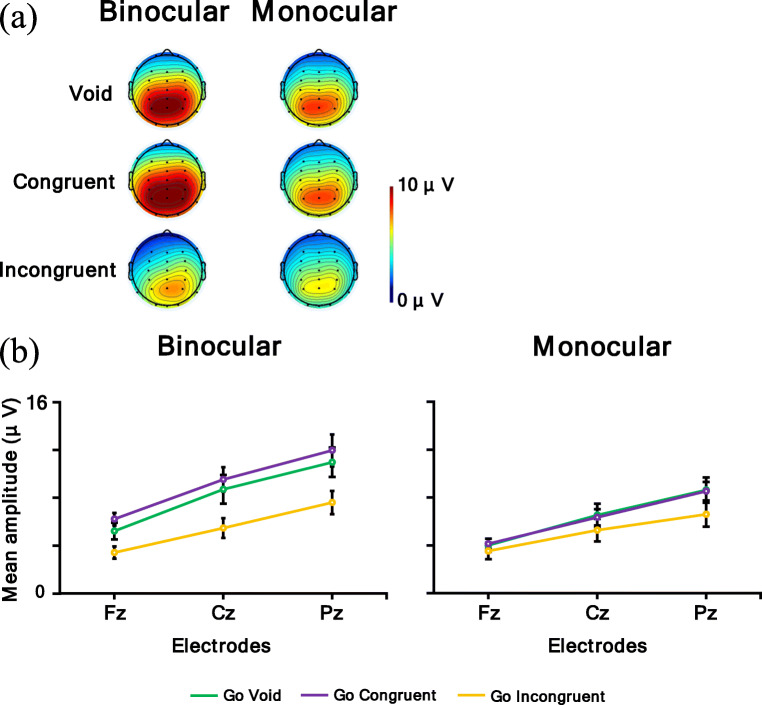
**a** Topographic maps for time range of N2 for Go condition (380–480 ms) and **b** N2 for Go condition mean amplitude. Error bars indicate standard error

Figure 9 shows grand averaged waves of the Go condition at Fz, Cz, and Pz, and Fig. 10 shows (a) a topographic map for the time range of N2 for the Go condition (380–480 ms) and (b) N2 for the Go condition mean amplitude. N2 is a negative potential; thus, the more negative the potential is, the larger the N2 response is. The ANOVA results are summarized in Table 5. It was revealed that the main effect of the flanker condition was significant. Post hoc comparisons showed that the mean amplitude of the incongruent condition was larger in the negative direction than in the void and congruent conditions (*p*s < .05). Moreover, the main effect of the electrodes was significant. Post hoc comparisons showed that Fz had the largest mean amplitude in the negative direction, followed in order by Cz and Pz (*p*s < .05). Furthermore, the main effect of the observation condition was significant, and the mean amplitude of N2 in the monocular condition was larger in the negative direction than the binocular condition (*p* < .05). In addition, the interaction of the flanker condition and electrodes was significant. Post hoc comparisons showed the same result for the main effect of the flanker condition and electrodes in the all electrode and flanker conditions (*p*s < .05). Finally, the interaction of the observation condition and electrodes was significant. Post hoc comparisons showed the same result for the main effect of the observation condition and electrodes in the all the electrode and observation conditions (*p*s < .05). In summary, N2 in the incongruent condition was elicited in both the binocular and the monocular conditions, and the amplitude of N2 at the frontal electrode was larger in the negative direction than at the central and parietal electrodes. In addition, the amplitude of N2 was different between the binocular and monocular conditions.

**Table. 5:** Analysis results of N2 for the Go condition

Interaction and main effect	*F*	*df*	*p*	η_p_^2^
observation × flanker × electrode	.64	4, 68	.55	.04
observation × flanker	2.62	2, 34	.10	.13
observation × electrode	7.75	2, 34	.009^**^	.31
flanker × electrode	6.51	4, 68	.003^**^	.28
Observation	11.17	1, 17	.004^**^	.40
Flanker	8.86	2, 34	.002^**^	.34
Electrode	59.72	2, 34	<.001^***^	.78

**Fig. 11:**
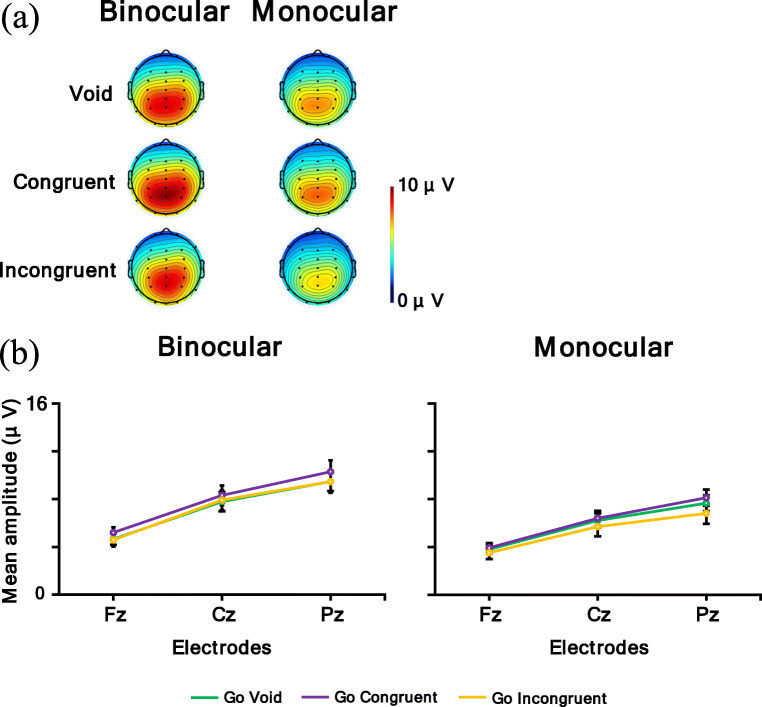
**a** Topographic maps for time range of P3 for Go condition (void and congruent conditions: 300–600 ms; incongruent condition in binocular condition: 460–660 ms) and **b** P3 for Go condition mean amplitude. Error bars indicate standard error

Figure 11 shows (a) a topographic map for the time range of P3 for the Go condition (void and congruent conditions: 300–600 ms; incongruent condition in binocular condition: 460–660 ms) and (b) P3 for the Go condition mean amplitude. In the P3 analysis, a larger amplitude means a larger P3 response. The ANOVA results are summarized in Table 6. It was revealed that the main effect of the observation condition was significant and the mean amplitude of the binocular condition was larger than the monocular condition (*p* < .05). Moreover, the main effect of the electrodes was significant. Post hoc comparisons showed that Pz had the largest mean amplitude, followed in order by Cz and Fz (*p*s < .05). Furthermore, the interaction of the observation condition and electrodes was significant. Post hoc comparisons showed the same result for the main effect of the observation condition and electrodes in all the electrode and observation conditions (*p*s < .05). In summary, the P3 amplitude elicited by the binocular condition was larger than the monocular condition, and the amplitude of P3 at the parietal electrode was larger than at the frontal and central electrodes.

**Table. 6:** Analysis results of P3 for the Go condition

Interaction and main effect	*F*	*df*	*p*	η_p_^2^
observation × flanker × electrode	.55	4, 68	.60	.03
observation × flanker	.27	2, 34	.71	.02
observation × electrode	13.52	2, 34	<.001^***^	.44
flanker × electrode	2.05	4, 68	.12	.11
Observation	38.54	1, 17	<.001^***^	.69
Flanker	1.51	2, 34	.24	.08
Electrode	83.96	2, 34	<.001^***^	.83

**Fig. 12:**
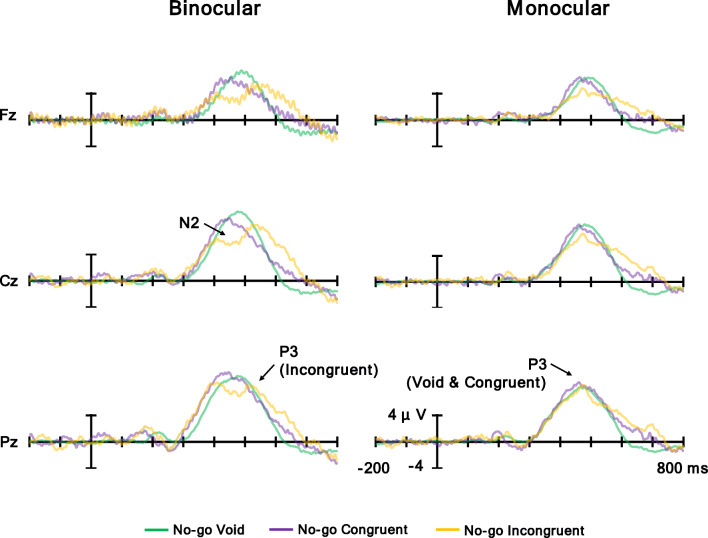
Grand averaged waves of No-go condition at Fz, Cz, and Pz

**Fig. 13:**
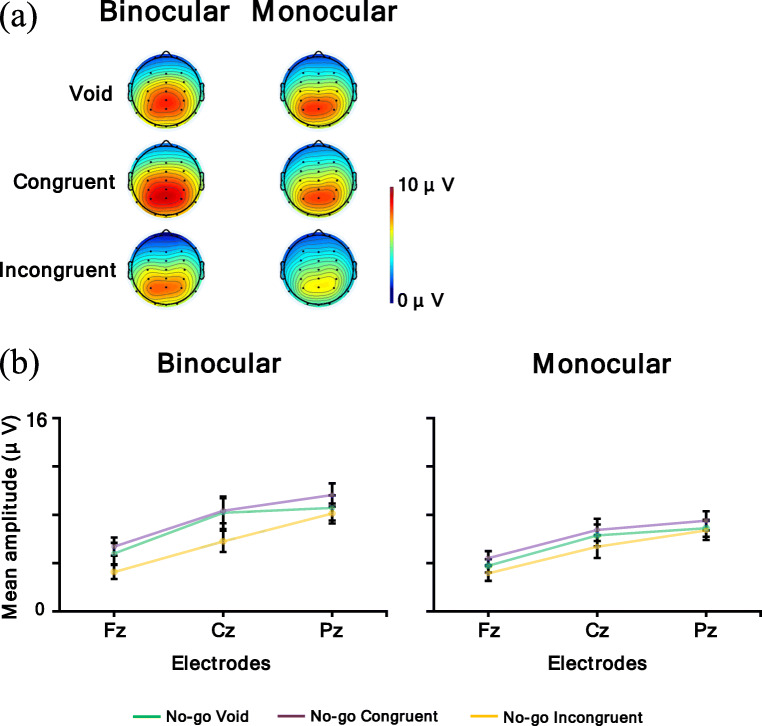
**a** Topographic maps for time range of N2 for No-go condition (380–480 ms) and **b** N2 for No-go condition mean amplitude. Error bars indicate standard error

Figure 12 shows the grand average waves of the No-go condition at Fz, Cz, and Pz, and Fig. 13 shows a (a) topographic map for the time range of N2 for the No-go condition (380–480 ms) and (b) N2 for the No-go condition mean amplitude. N2 is a negative potential; thus, the more negative the potential is, the larger the N2 response is. The ANOVA results are summarized in Table 7. It was revealed that the main effect of the flanker condition was significant. Post hoc comparisons showed that the mean amplitude of the incongruent condition was larger than the void and congruent conditions (*p*s < .05). Moreover, the main effect of the electrodes was significant. Post hoc comparisons showed that Fz had the largest mean amplitude, followed in order by Cz and Pz (*p*s < .05). Furthermore, the interaction of the flanker condition and electrodes was significant. Post hoc comparisons showed the same result for the main effect of the flanker condition at Fz and Cz and the electrodes in the congruent and incongruent conditions (*p*s < .05). In the void condition, the mean amplitude of N2 at Fz was larger than at Cz and Pz (*p*s < .05). Finally, the interaction of the observation condition and electrodes was significant. Post hoc comparisons showed the same result for the main effect of the electrodes in all observation conditions (*p*s < .05). For Pz, the mean amplitude of the monocular condition was larger than the binocular condition (*p*s < .05). In summary, the incongruent condition elicited N2 in both conditions, and the amplitude of N2 at the frontal electrode was larger than the central and parietal electrodes.

**Table. 7:** Analysis results of N2 for the No-go condition

Interaction and main effect	*F*	*df*	*p*	η_p_^2^
observation × flanker × electrode	1.51	4, 68	.23	.08
observation × flanker	.82	2, 34	.43	.05
observation × electrode	8.11	2, 34	.002^**^	.32
flanker × electrode	4.43	4, 68	.01^**^	.21
Observation	4.36	1, 17	.06	.20
Flanker	4.41	2, 34	.01^**^	.21
Electrode	40.97	2, 34	<.001^***^	.71

**Fig. 14:**
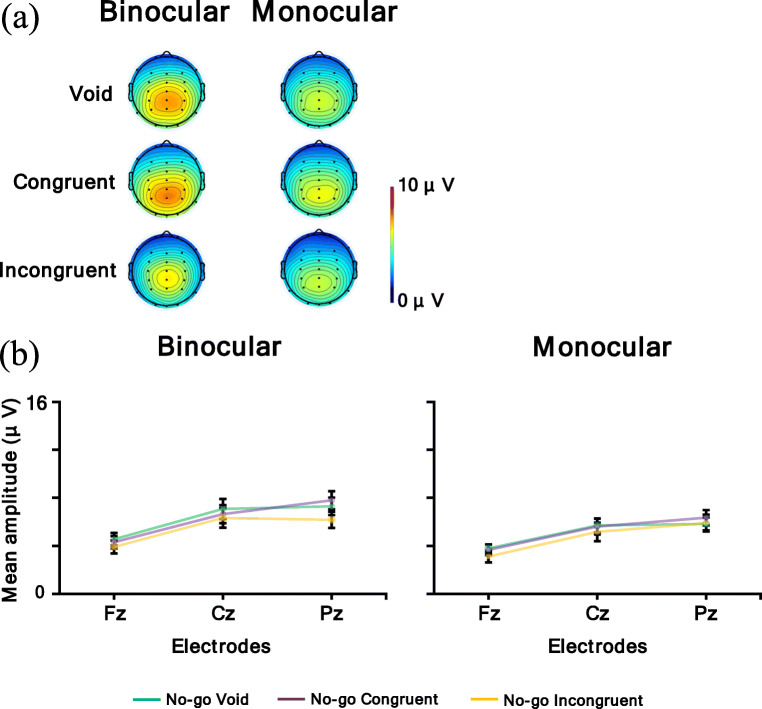
**a** Topographic maps for time range of P3 for the No-go condition (void and congruent condition: 300–600 ms; incongruent condition in binocular condition: 460–660 ms) and **b** P3 for the No-go condition mean amplitude. Error bars indicate standard error

Figure 14 shows a (a) topographic map for the time range of P3 for the No-go condition (void and congruent condition: 300–600 ms; incongruent condition in the binocular condition: 460–660 ms) and (b) P3 for the No-go condition mean amplitude. In the P3 analysis, a larger amplitude means a larger P3 response. The ANOVA results are summarized in Table 8. It was revealed that the main effect of the observation condition was significant, and the mean amplitude of the binocular condition was larger than the monocular condition (*p* < .05). Moreover, the main effect of the electrodes was significant. Post hoc comparisons showed that the mean amplitude of Pz and Cz were larger than Fz (*p*s < .05). Furthermore, the interaction of the flanker condition and electrodes was significant. Post hoc comparisons showed the same result for the main effect of the electrodes in the void and incongruent conditions (*p*s < .05). In the congruent condition, Pz had the largest mean amplitude, followed in order by Cz and Fz (*p*s < .05).

**Table. 8:** Analysis results of P3 for the No-go condition

Interaction and main effect	*F*	*df*	*p*	η_p_^2^
observation × flanker × electrode	3.56	4, 68	.03^*^	.17
observation × flanker	.25	2, 34	.73	.01
observation × electrode	2.16	2, 34	.14	.11
flanker × electrode	4.61	4, 68	.01^*^	.21
Observation	7.12	1, 17	.02^*^	.30
Flanker	2.00	2, 34	.15	.11
Electrode	48.37	2, 34	<.001^***^	.74

In addition, the interaction of the observation condition, flanker condition, and electrodes was significant. Table 9 shows the simple interaction effects and simple-simple main effects.

**Table. 9:** Simple interaction and simple-simple main effect of P3 for the No-go condition

Simple interaction and simple-simple main effect	*F*	*df*	*P*	η_p_^2^
Binocular condition				
flanker × electrode	4.63	4, 68	.01^*^	.21
flanker	1.73	2, 34	.20	.09
electrode	45.91	2, 34	<.001^***^	.73
Monocular condition				
flanker × electrode	2.98	4, 68	.06	.15
flanker	.60	2, 34	.55	.03
electrode	37.82	2, 34	<.001^***^	.69
Void condition				
observation × electrode	7.92	2, 34	.002^**^	.32
observation	10.24	1, 17	.005^**^	.38
electrode	33.89	2, 34	<.001^***^	.67
Congruent condition				
observation × electrode	3.16	2, 34	.08	.16
observation	3.10	1, 17	.10	.15
electrode	48.28	2, 34	<.001^***^	.74
Incongruent condition				
observation × electrode	1.94	2, 34	.17	.10
observation	1.58	1, 17	.23	.08
electrode	43.41	2, 34	<.001^***^	.72
Fz				
observation × flanker	.06	2, 34	.91	.01
observation	4.56	1, 17	<.05^*^	.21
flanker	1.95	2, 34	.16	.10
Cz				
observation × flanker	.10	2, 34	.86	.01
observation	7.01	1, 17	.02^*^	.29
flanker	1.24	2, 34	.30	.07
Pz				
observation × flanker	1.54	2, 34	.23	.08
observation	7.49	1, 17	.01^*^	.31
flanker	3.75	2, 34	.04^*^	.18

For the factor of binocular condition, the simple interaction of the flanker condition and electrodes was significant. Post hoc comparisons showed that the mean amplitudes of Pz and Cz were larger than Fz in the void and incongruent conditions (*p*s < .05). In the congruent condition, Pz had the largest mean amplitude, followed in order by Cz and Fz (*p*s < .05). In addition, the simple-simple main effect of the electrodes was significant, and the mean amplitudes of Pz and Cz were larger than Fz (*p*s < .05). For the factor of the monocular condition, the simple-simple main effect of the electrodes was significant. Post hoc comparisons showed that Pz had the largest mean amplitude, followed in order by Cz and Fz (*p*s < .05).

For the factor of the void condition, the simple interaction of the observation condition and electrodes was significant. Post hoc comparisons showed that the mean amplitude of the binocular condition was larger than the monocular condition for all electrodes (*p*s < .05). Pz had the largest mean amplitude, followed in order by Cz and Fz in the binocular and monocular conditions (*p*s < .05). In addition, the simple-simple main effect of the observation condition was significant, and the mean amplitude of the binocular condition was larger than the monocular condition (*p* < .05). Additionally, the simple-simple main effect of the electrodes was significant. Post hoc comparisons showed that the mean amplitudes of Pz and Cz were larger than Fz (*p*s < .05). For the factor of congruent condition, the simple-simple main effect of the electrodes was significant. Post hoc comparisons showed that Pz had the largest mean amplitude, followed in order by Cz and Fz (*p*s < .05). For the factor of incongruent condition, the simple-simple main effect of the electrodes was significant. Post hoc comparisons showed that the mean amplitudes of Pz and Cz were larger than Fz (*p*s < .05).

For the electrodes of Fz, Cz, and Pz, the simple-simple main effect of the observation condition was significant, and the mean amplitude of the binocular condition was larger than the monocular condition (*p*s < .05).

In summary, the P3 amplitude at the parietal electrode was larger than the frontal and central electrodes, and the mean amplitude of the binocular condition was larger than the monocular condition. Moreover, the interaction revealed this observation effect in the void condition.

### Subjective data analysis

In the present study, participants answered questions as a subjective measure after each observation block (see Table 1).

For Q1, the subjective attentional control was measured, and the mean was 5.70 (*SD* = 2.39).

Q2 and Q4 were questions for measuring the subjective perception of the flanker stimuli in both the monocular and binocular conditions. Hence, a paired *t* test was conducted. The result revealed that the flanker stimuli were more perceived in the binocular condition (*mean* = 8.55, *SD* = 2.06) than in the monocular condition (*mean* = 6.40, *SD* = 3.33) (*t* (17) = 3.00, *p* = .008^**^).

Q3 and Q5 were questions for measuring the subjective annoyance of the flanker stimuli in both the monocular and binocular conditions. Hence, a paired *t* test was conducted. The result revealed that the subjective annoyance of the flanker stimuli was not significantly different between the binocular (*mean* = 5.40, *SD* = 2.62) and monocular conditions (*mean* = 5.00, *SD* = 3.16) (*t* (17) = 0.15, *p* = .882).

## Discussion

In the present study, the difference between monocular and binocular AR presentation in information processing was investigated by using a task that combined the flanker task with the oddball task.

The results for the reaction times (Figs. 4 and 5 and Table 2) revealed that the reaction times were shorter in the binocular condition than in the monocular condition generally; however, in only the incongruent condition, they were shorter in the monocular condition. Both congruent and incongruent flankers made reaction times longer compared with the void condition, regardless of the observation conditions. The results for the hit rates (Fig. 6 and Table 3) revealed that the monocular condition led to lower performance than the binocular condition, especially in the incongruent condition. These behavioral results revealed that the flanker stimuli had influence not only in the binocular condition but also in the monocular condition; therefore, the hypothesis that the flanker stimuli would not be processed in the monocular condition was not fully supported.

However, in the incongruent condition, the influence of the flanker stimuli was smaller in the monocular condition than in the binocular condition; at the least, for this point, the result partially supported the hypothesis. A histogram of the reaction times (Fig. 5) revealed that the distribution was wider in the monocular condition than in the binocular condition, meaning that participants responded earlier in some (not all) trials compared with the binocular condition.

This result could suggest that the flanker stimuli are processed less in the monocular condition, though not perfectly ignored. For example, in one trial the effect of the incongruent flanker stimuli was very strong, and in another trial, the effect becomes weak; in this way, the effect of flankers might be different among each trial. The subjective data also revealed that the perception of the flanker stimuli was weaker in the monocular condition than in the binocular condition, but the stimuli were not perfectly ignored. In addition, previous studies revealed that binocular rivalry is influenced by endogenous attention; however, observers cannot perfectly control their perception in binocular rivalry by their own will (Chong et al., 2005; Chong & Blake, 2006; Hancock & Andrews, 2007; Meng & Tong, 2004; Ooi & He, 1999; Paffen & Alais, 2011; Zhang et al., 2012). Even though the initial percept and its duration in binocular rivalry is influenced by an observer’s endogenous attention, inevitably, it changes at a certain probability. These facts support the above explanation.

However, we do not have enough evidence to conclude that this explanation is correct. Hence, more precise scrutiny and replication studies should be conducted to confirm how robust the phenomenon is.

Overall, the behavioral performance declined in the monocular condition. Qian et al. (2019) reported that, among most AR equipment, users preferred binocular AR systems to monocular ones because binocular rivalry annoyed users in the case of monocular AR. Thus, even though monocular AR has some advantage (Kitamura et al., 2014, 2015, 2019), it might depend on the characteristics of the task.

Used as a physiological measure, ERP distributions were similar between the monocular and binocular conditions, and vMMN, N2, and P3s were elicited in both conditions. The results for vMMN reveal that the flanker stimuli elicited vMMN in both conditions (Fig. 8 and Table 4). vMMN is caused by a flanker stimulus deviating from a stimulus sequence (i.e., distractor) in a flanker task (Noyce & Sekuler, 2014) and reflects the pre-attentional stage (Stefanics et al., 2011). This result means that a similar vMMN being elicited in monocular and binocular observations could indicate that the processing of the visual image did not differ at the pre-attentional stage. Moreover, the amplitude for the Go condition was larger than the No-go condition in the monocular condition. This result suggests that the degree of deviation of the oddball between target and non-target might be different in the monocular condition. It would be possible to examine this difference by using a three-stimulus oddball task (e.g., Katayama & Polich, 1996, 1998). In this task, a high-frequency standard stimulus, low-frequency target stimulus (Go stimulus), and low-frequency deviant stimulus (No-go stimulus) are presented to participants. Participants should not respond to deviant stimuli, but deviant stimuli attract involuntary attention and elicit MMN and P3a. It would be possible to simply examine how much the monocular condition suppresses irrelevant stimuli (deviant stimulus) by comparing binocular and monocular conditions by presenting deviant stimuli on an AR display. It is necessary to examine this point in the future.

N2 at the frontal electrode in both target conditions was evoked by incongruent flanker stimuli in both the binocular and the monocular conditions (Figs. 10 and 13, Tables 5 and 7). For the Go N2, the amplitude of N2 was different between the binocular and monocular conditions; however, this result was not specific to the N2 by the incongruent flanker stimuli. That is, the difference in amplitude among the incongruent condition and other conditions was smaller in the monocular condition. N2 is caused by flanker stimuli in the incongruent condition and reflects conflict processing (Kopp, Mattler, et al., 1996a; Kopp, Rist, & Mattler, 1996b). Therefore, the congruency of the flanker stimuli was processed in both observation conditions, revealing that the meaning of the flanker stimuli was processed regardless of the observation conditions.

In addition, both the Go P3s at the parietal electrode and the No-go P3 at the central-parietal electrode were larger in the binocular condition than in the monocular condition. Go P3s, caused by Go stimuli in the frontal to central regions (i.e., Fz to Cz electrodes), were evoked for the target, and the No-go P3s caused by No-go stimuli in the central to parietal regions (i.e., Cz to Pz electrodes) were evoked for non-targets (Pfefferbaum et al., 1985; Pfefferbaum & Ford, 1988). These P3s reflect the stimulus evaluation (Callaway, 1983; Duncan et al., 2009; Verleger, 1997, 2010), and the No-go P3 was related to inhibition (Pfefferbaum et al., 1985) and response conflict (Smith et al., 2010). Therefore, these results imply that for both reaction and inhibition, participants distributed more attention to the target in the binocular condition than in the monocular condition, and there were some differences between the two conditions at the cognitive process stage. Several reasons might be possible for explaining why participants distributed attention less to the task in the monocular condition. One reason might be because, in the monocular condition, participants were instructed to focus on information from the eye to which the AR images were not displayed, so they might have had to apply attentional resources to following the instruction. The other reason might be because binocular rivalry would occur in the monocular condition, and binocular rivalry itself was distracting, so participants might not have been able to concentrate on the task. Participants might have had to deal with these issues; thus they could not distribute attention to the target as sufficiently as in the binocular condition. Therefore, it might be thought that this contribution influenced the cognitive process stage and that the amplitudes of P3s were different between the binocular and monocular conditions. This explanation is consistent with the results for the reaction times, which were longer in the monocular condition generally. Furthermore, in the incongruent condition, the latencies of P3s were delayed in the binocular condition compared with the other flanker conditions, but there was no obvious delay in the monocular condition, and the distribution of amplitude became wider. This result might be because, in the binocular condition, a P3 is more affected by the immediately preceding N2, that is, the competitive reaction to the incongruent flanker stimuli was large. In the monocular condition, it is possible that the influence of the flanker stimuli was not the same in each trial as mentioned above, so the effect of the flankers might become ambiguous. Therefore, the results of the physiological measure were consistent with those of the behavioral measures.

However, some results revealed that the difference between the monocular and binocular conditions was not significant, and binocular rivalry itself was not investigated in the present study; therefore, these explanations above were only consistent with the results of the present study. More precise scrutiny is needed in a future study as well as behavioral measures.

To summarize the results of the physiological measure, the flanker stimuli were processed in both perception and meaning, regardless of the observation condition. It was revealed that participants could not control their perception perfectly by their own will even in the monocular condition, and the results did not support our hypothesis that participants could ignore information from the eye to which AR flanker stimuli were presented in the perceptual stage.

Putting these results together, at the pre-attentional stage, the flanker information was processed in the monocular condition as well as the binocular condition, and endogenous attention could not control perception perfectly in the monocular condition. However, the stimuli did not always influence the reaction times. Therefore, a stochastic mechanism might influence the perception of stimuli in the monocular condition. This mechanism might be related to the subjective percept in binocular rivalry; however, this was not investigated in the present study, so more precise scrutiny is needed regarding this mechanism.

## Conclusion

In the present study, we investigated whether the influence of information that was not required for a task could be attenuated in monocular AR compared with binocular AR presentation. The results revealed that participants could not ignore the flanker stimuli even in the monocular condition. However, the influence of the stimuli was unstable in the monocular condition compared with the binocular condition; as a result, distraction from the stimuli was less in the monocular condition. At the pre-attentional (i.e., vMMN) and stimulus conflict stage (i.e., N2), the physiological measure revealed that the flanker stimuli were processed both in the monocular and binocular conditions. However, the measures reflecting the reaction process, such as the P3s, reaction times, and hit rates, were different between the two conditions, and this implies that participants might have been able to distribute more attention to the task in the binocular condition than in the monocular condition. The mechanism that influences these reactions was not clarified in the present study, so more precise scrutiny is needed.
